# Investigation of the effects of monosodium glutamate and tannic acid on the glutathione and thioredoxin systems in the liver of rats

**DOI:** 10.1007/s00210-025-04279-5

**Published:** 2025-05-21

**Authors:** Mohammad A. A. Abushikha, Medine Sibel Karagac, Esra Nur Yesilkent, Hamid Ceylan

**Affiliations:** 1 Department of Molecular Biology and Genetics, Faculty of Science, Atatürk University, 25240 Erzurum, Türkiye; 2 East Anatolian High Technology Research and Application Center (DAYTAM), Atatürk University, 25240 Erzurum, Türkiye

**Keywords:** Monosodium glutamate, Hepatotoxicity, Glutathione, Thioredoxin, Tannic acid

## Abstract

While there is no conclusive evidence that monosodium glutamate (MSG, a food additive) directly causes liver cancer in humans, certain studies suggest a potential link between MSG-induced liver injury and cancer development. This study aimed to evaluate the protective effect of tannic acid (TA, a natural polyphenol) against MSG-induced hepatotoxicity through the glutathione and thioredoxin systems. Twenty-four rats were randomly divided into control and experimental groups and treated with TA, MSG, and MSG+TA once daily by oral gavage for 21 days. In addition to major oxidative stress indicators (total glutathione; GSH + GSSG and malondialdehyde; MDA), mRNA expression changes and biological activity responses of components of the glutathione and thioredoxin systems were examined in the liver tissues of all animals. The results showed that MSG alone negatively affected both stress indicators and antioxidant system components (glutathione peroxidase; GPx, glutathione reductase; GR, glutathione-S-transferase; GST, and thioredoxin reductase; TrxR) in terms of mRNA expression and biological activity. However, the combination of MSG and TA demonstrated robust antioxidative effects, surpassing the outcomes of MSG treatment. Our results provide new insights into pivotal molecular targets and protective candidates that should be focused on in future in vivo and in vitro HCC research.

## Introduction

Monosodium glutamate (MSG) is a form of glutamic acid, one of the 20 natural amino acids. MSG is a food additive widely used in processed foods or fast food products due to its flavor-enhancing properties (Sukmak et al. 2024; Thongsepee et al. 2024). While regulatory bodies such as the US Food and Drug Administration (FDA) have classified MSG as generally recognized as safe (GRAS), ongoing scientific debate has raised concerns about its potential health effects (Faustman et al. 2021). Recent studies have focused on the long-term consequences that MSG may cause beyond its short-term effects, defined as the MSG symptom complex (Shastri et al. 2023), particularly on liver health (Onaolapo et al. 2016; Shimada et al. 2015). Studies have suggested that MSG significantly increases the levels of critical enzymes that are indicators of liver damage, such as aspartate aminotransferase (AST) and alanine transaminase (ALT), and may cause hepatotoxicity (Banerjee et al. 2021; Sahin et al. 2023). It is also thought that MSG may exacerbate the damage by exhibiting a synergistic effect with other factors, such as lipopolysaccharide (LPS) that can cause liver damage (Asejeje et al. 2023). Given the growing body of literature suggesting a link between MSG and liver dysfunction, there is a critical need for further investigation into the underlying mechanisms of MSG-induced toxicity. Such research could also aid in identifying potential therapeutic agents capable of mitigating these effects.

Polyphenolic compounds, a group of phytochemicals found in many plants, have attracted significant interest in the medical field due to their numerous benefits (Karagaç et al. 2024; Kumar et al. 2023). In addition to their biological properties, their cost-effectiveness and sustainability make these compounds, which include phenolic acids, flavonoids, and tannins, more attractive (Aatif 2023). As research continues, exploring the molecular mechanisms underlying protective effects and the therapeutic potential of natural polyphenolic compounds in medicinal applications is becoming increasingly evident (Karadas et al. 2024). Tannic acid (TA), which can be found in many plants, is the simplest hydrolyzable tannin and is a US Food and Drug Administration (FDA)-approved food additive (Guo et al. 2021). The hydroxyl groups of plant-based polyphenols, such as TA, provide these compounds with hydrogen donor and metal chelating properties, thus making these compounds suitable candidates against oxidative stress-related abnormalities (Varesi et al. 2022). TA is known to have greater antioxidant potential (Dare et al. 2020; Kizir et al. 2023, 2024), superior antibacterial and anti-inflammatory properties (Sahiner et al. 2023), and superior bioavailability and bioaccessibility (Fraga-Corral et al. 2021) compared to other flavonoids, such as gallic acid. Moreover, it has attracted considerable attention in biomaterial development studies because of its ability to form numerous hydrogen bond interactions with a wide variety of molecules (Baldwin & Booth 2022). Such extensive physiological and chemical properties make TA a very strong natural candidate. Recent studies have suggested that TA tends to have a similar impact trend in healing various types of damage that can occur in the liver as a result of different factors (Chu et al. 2016; Yesilkent & Ceylan 2022; Zhang et al. 2017). Research indicates that, by enhancing the cellular antioxidant defense capacity and scavenging free radicals, TA effectively combats MSG-induced oxidative stress (Karagac & Ceylan 2023). Studies using experimental animal models have shown that hepatic enzymes such as AST and ALT and lipid peroxidation are reduced, and glutathione levels are restored with TA (Li et al. 2020; Ozturk et al. 2024). Additionally, the production of pro-inflammatory cytokines that cause hepatic inflammation and fibrosis has also been proven to be reduced in the presence of TA (Chu et al. 2016; Lin et al. 2023). Moreover, by modulating apoptotic pathways, TA exhibits protective effects and supports cell survival mechanisms (Wang et al. 2019). All these reports show that the structural integrity of hepatocytes can be preserved by preventing the factors that cause liver damage with TA. Thus, we hypothesized that these versatile actions position TA as a potent prophylactic agent against MSG exposure-induced alterations in liver tissue.

In the present study, it is aimed to elucidate the molecular mechanisms underlying the protective effects of TA and shed light on potential future therapeutic applications. First, the effects of MSG and TA on healthy rat liver were examined. Subsequently, MSG-induced liver injury model rats were established, and the protective effects of TA on thioredoxin and glutathione systems were investigated.

## Materials and methods

### Animals and ethics approval

Twenty-four rats (*Rattus norvegicus*, Sprague–Dawley, male, 180 g ± 10 g) were obtained from the Atatürk University Medical Experimental Application and Research Center (Erzurum, Turkey). The Atatürk University Animal Experiments Local Ethics Committee approved this experimental protocol (protocol no.: 2021–3/63), and all experimental procedures were conducted in compliance with the NIH (National Research Council Committee, 2011) Guide for the Care and Use of Laboratory Animals. Additionally, to improve transparency, the study design and experiments were reported following ARRIVE 2.0 (Animal Research: Reporting of In Vivo Experiments, 2020) guidelines and the 3R/6R principles.

### Experimental protocol

**Fig. 1:**
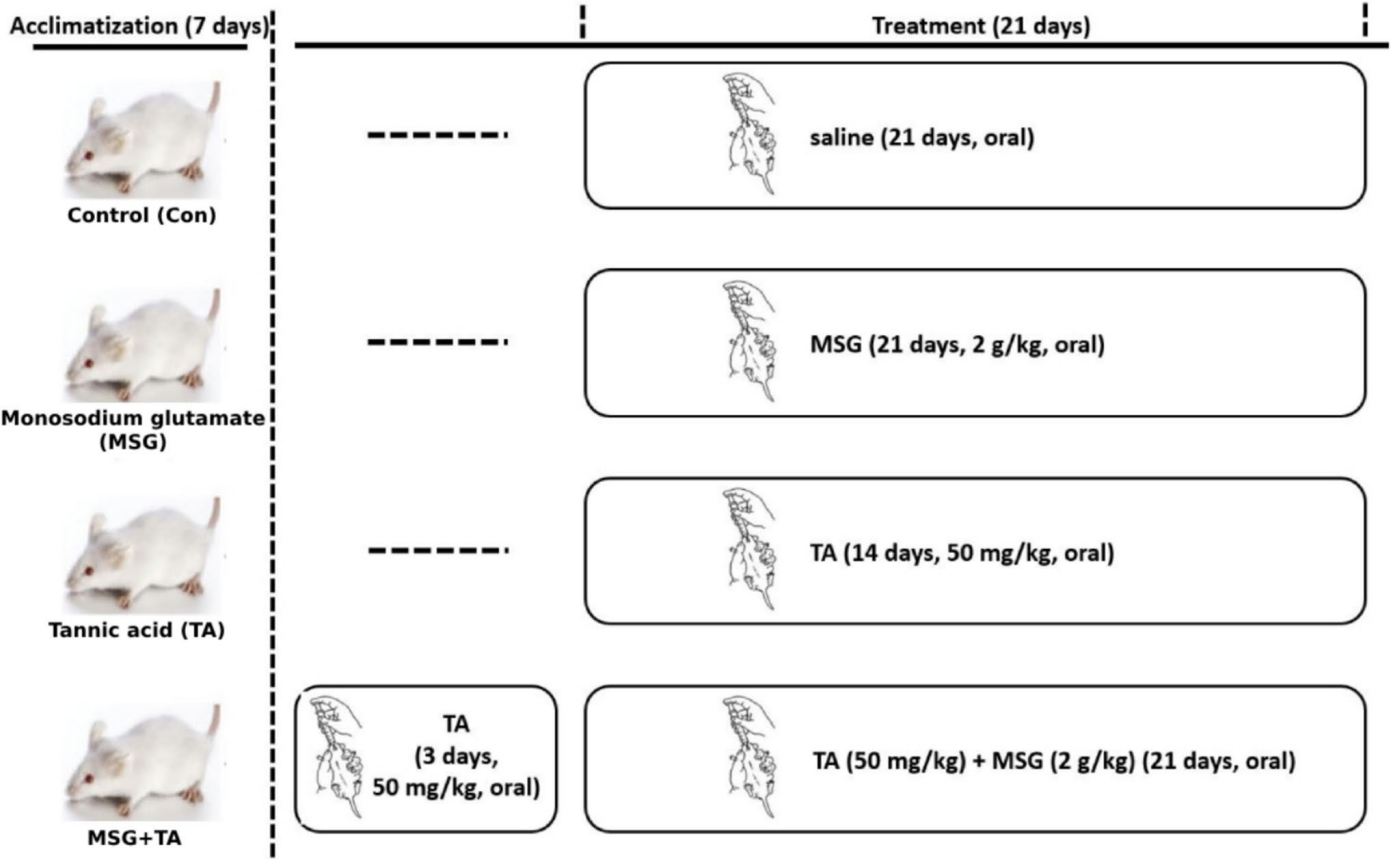
Experimental protocol of the study

After an acclimatization period, rats were randomly divided into four equal groups (Con, control; MSG, monosodium glutamate; TA, tannic acid; and MSG + TA, combination of monosodium glutamate and tannic acid), each consisting of six animals (Fig. 1). The control group rats were treated with a vehicle (normal saline) for 21 days. Monosodium glutamate (≥ 98.0% purity, Sigma-Aldrich, cat. no. 49621) and tannic acid (≥ 95.0 purity, Thermo Scientific Chemicals, cat. no. 202420050), dissolved in the vehicle and prepared daily, were administered by oral gavage once daily for 21 days at 2 g/kg and 50 mg/kg, respectively. Previous reports indicate that the average daily intake (ADI) of monosodium glutamate (MSG) is around 10 g/day, which is generally considered to have an optimal safety profile. Furthermore, 16.0 mg/kg of body weight consumption has been identified as the no observed adverse effect level (NOAEL) (Beyreuther et al. 2007). Despite the recommended daily limit for adult MSG intake being less than 6 g/day (Thongsepee et al. 2022), several in vivo studies have shown that even lower doses can result in harmful effects, including cardiotoxicity (Hazzaa et al. 2020), renal toxicity (Koohpeyma et al. 2021), neurotoxicity (Hazarika et al. 2022), and hepatotoxicity (Omogbiya et al. 2021). Therefore, rats in the MSG group were orally exposed to 2 g/kg (lower than ADI and NOAEL) of MSG. TA dose was also chosen based on previous research (Biney et al. 2022; Tüzmen et al. 2015). MSG and TA were applied simultaneously to the combined group (MSG + TA) animals for 21 days. To improve the prophylactic effect of the TA, it was administered to the rats in the combined group 1 h before MSG (Al-Jaouni et al. 2019). All groups were housed in plastic cages under standard conditions (free access to diet and tap water, etc.). The rats were euthanized under ketamine/xylazine (3:1) anesthesia, and liver tissues were immediately removed and washed with cold phosphate-buffered saline and kept at − 80 °C for subsequent studies.

### Assessment of oxidative stress indicators

To examine the oxidative stress status after MSG exposure and TA treatment in the liver tissues of untreated and other experimental rat groups, malondialdehyde (MDA; secondary products of lipid peroxidation) levels and total glutathione (GSH + GSSG) contents, which are biomarkers of oxidative stress, were measured. MDA levels in rat liver tissue were measured at wavelengths of 532 nm according to the thiobarbituric acid method as described previously (Suleyman et al. 2009) and presented as nanomoles MDA per mg protein. The reduced glutathione (GSH) quantity in tissue samples was measured at wavelengths of 450 nm as recently described (Kocpinar et al. 2020).

### Measurements of the enzyme activities

Total protein was determined by the Bradford method (Bradford 1976) using BSA (bovine serum albumin, ≥ 98.0% purity, Sigma-Aldrich, cat. no. A7906, 1 mg/mL) as a standard. To measure glutathione peroxidase (GPx) enzymatic activity, 100 mg of liver tissues were homogenized (Heidolph Silent Crusher M, Germany) in a buffer containing 1 mM EDTA (ethylenediaminetetraacetic acid, ≥ 98.0% purity, Sigma-Aldrich, cat. no. 798681), 1 mM DTT (dithiothreitol, Sigma Supelco, cat. no. 49018), 1 mM PMSF (phenylmethylsulfonyl fluoride, Roche, cat. no. 10837091001), and 50 mM Tris HCl (Yesilkent & Ceylan 2022). To measure glutathione S-transferase (GST) enzymatic activity, the optical density of 10 µL of supernatant of liver tissue homogenate, 20 mM GSH, and 100 mM phosphate buffer contained mixture was measured at 340 nm after adding 6 mM NADP^+^ (β-nicotinamide adenine dinucleotide phosphate disodium salt, Roche, cat. no. 10128031001) (Oztay et al. 2020). Glutathione reductase (GR) activity was measured by the modified method of Carlberg and Mannervik (Carlberg & Mannervik 1985) as previously described (Budak et al. 2014). Thioredoxin reductase (TrxR) activity measurement was performed by modifying the method developed by Arner and Holmgren (Arner & Holmgren 2006) as previously described (Kansu et al. 2024).

### Quantitative real-time PCR (qPCR) analysis

For relative quantification of target genes mRNA expression, firstly, total RNA was extracted from the rat liver using a commercial total RNA extraction kit (Biorad, Hercules, CA, USA) following the manufacturer’s instruction. Then, the cDNA library was constructed using the iScript cDNA synthesis kit (Biorad, cat. no. 1708891) following the manufacturer’s recommendations. To detect gene expression, pairs of specific primers (Table 1) were designed using the Primer3 (https://bioinfo.ut.ee/primer3-0.4.0/, accessed on 15 June 2024) online tool (Untergasser et al. 2012). The binding specificities of the determined primer sequences were confirmed using the https://blast.ncbi.nlm.nih.gov/Blast.cgi (accessed on 15 June 2024) module. For relative quantification, the SYBR Green-based qPCR assay was performed using SsoAdvanced™ Universal SYBR® Green Supermix (Biorad, cat. no. Biorad, cat. no. 1708891). The 10 µL PCR reaction volume contained cDNA, forward and reverse primers (250–500 nM each), and 1X SsoAdvanced™ Universal SYBR ® Green Supermix (2 ×). Polymerase activation and DNA denaturation occurred for 2 min at 95 °C, amplification for 40 cycles, with denaturation for 15 s at 95 °C, and annealing and extension at 60 °C for 20 s. *Gapdh* (NM_017008.3) was used as a housekeeping control. The comparative ΔΔCt method (Livak & Schmittgen 2001) was used for the relative quantification of gene expression.

**Table 1:** Primer sets used in qPCR. F, forward; R, reverse; Tm, melting temperature

Gene symbol	Accession ID	Sequence	Tm (°C)
*Gpx_F* *Gpx _R*	NM_030826.4	5′-TCGGACATCAGGAGAATGG-3′	59.57
		5′-AGGTAAAGAGCGGGTGAGC-3′	59.44
*Gst_F* *Gst_R*	NM_001010921.1	5′-TTCTGACCCCTTTCCCTCTG-3′	59.67
		5′-TGGCTGGCTTTCTCTGACTG-3′	59.97
*Gr_F* *Gr_R*	NM_031632.1	5′-TGTGGTGGTGAGCAGAAAGA-3′	60.26
		5′-TCCTGGTATGGGACAGCATC-3′	59.95
*Txnrd_F* *Txnrd_R*	NM_022584.3	5′-AAGCCGTGCAAAACCATGTG-3′	59.97
		5′-ACCGTGAACTGTGTGCTCGT-3′	60.04
*Gapdh_F* *Gapdh_R*	NM_017008.3	5′-AAACCCATCACCATCTTCCA-3′	60.17
		5′-ATACTCAGCACCAGCATCACC-3′	60.16

### Statistical analysis

Statistical comparison of data obtained from measurements made in triplicate (for each animal and sample) was evaluated with one-way ANOVA and Tukey’s post hoc test using Prism (GraphPad Software, San Diego, CA) software. Significant difference between groups (compared to the control group) are indicated with asterisks. The statistically significant differences are presented as follows: ^ns^*p* > 0.05 (not significant), *p < 0.05 (significant), **p < 0.01 (very significant), *** or ****p < 0.001 or 0.0001 (extremely significant).

## Results

### Effects of MSG and TA administration on liver MDA and total GSH content

**Fig. 2:**
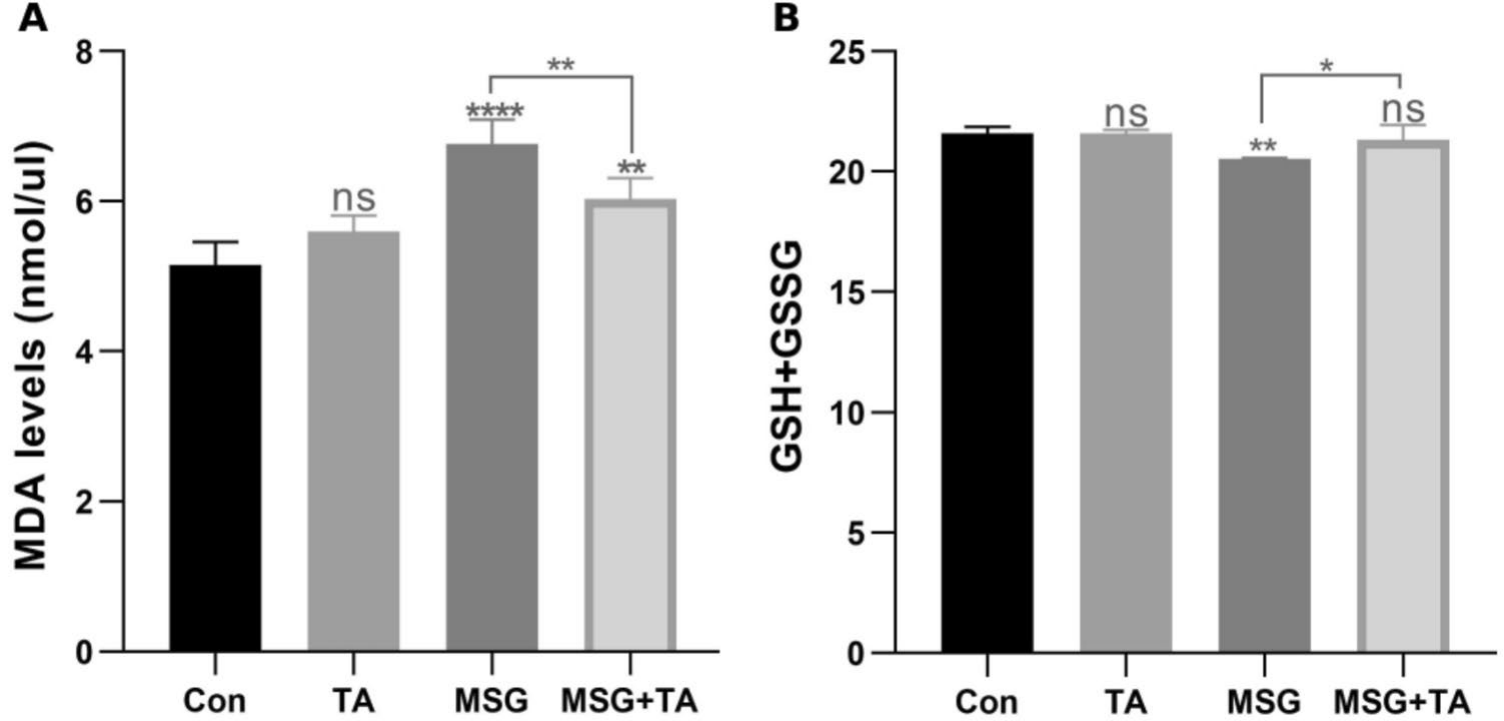
Malondialdehyde (MDA) levels and total glutathione (GSH + GSSG) content in the rat liver tissues. MDA levels in liver tissues (**A**) and comparison of total GSH content (**B**). ns represents *p* > 0.05, *p < 0.05, **p < 0.01, ***p < 0.001, and ****p < 0.0001 vs control group. The data are shown as mean ± SEM (*n* = 5)

To explore the effects of MSG and TA on the redox balance in rat liver, lipid peroxidation and total glutathione contents were analyzed. First, the concentration of MDA was measured in the livers of rats treated with MSG, both alone and combined with TA. As shown in Fig. 2A, a significant increase in MDA levels was observed in the livers of rats in the MSG-only group compared to the control group. In contrast, TA administration alone did not result in a significant increase in MDA levels. Additionally, TA effectively reduced the increase in lipid peroxidation induced by MSG. The results presented here clearly show that the MSG exposure may cause lipid peroxidation, which is induced by an excess of ROS in the rat liver. When examining GSH levels, it was found that MSG treatment alone significantly decreased total GSH content, while TA alone did not have an effect on GSH levels (Fig. 2B). Moreover, the simultaneous administration of TA with MSG reversed the reduction in GSH levels caused by MSG. Our results have evidence of the disruption of glutathione homeostasis after MSG exposure. However, the results presented here revealed that TA treatment enabled the stabilization of glutathione depletion.

### Glutathione system response in liver tissue after MSG and TA administration

**Fig. 3:**
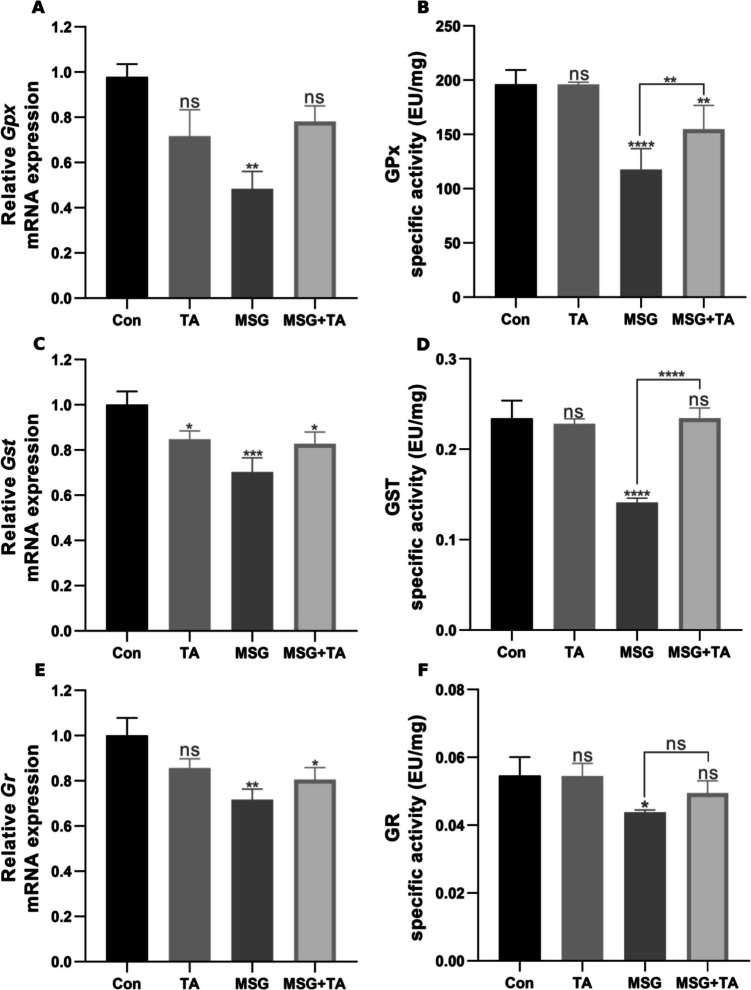
Effects of MSG and TA on the mRNA expression and specific activities of glutathione metabolism members in the rat liver tissues. The relative mRNA expressions of *Gpx*, glutathione peroxidase (**A**), *Gst*, glutathione S-transferase (**C**), and *Gr*, glutathione reductase (**E**). The enzymatic activities of GPx (**B**), GST (**D**), and GR (**F**) after saline and TA, MSG, and MSG + TA treatment. ns represents *p* > 0.05, *p < 0.05, **p < 0.01, ***p < 0.001, and ****p < 0.0001 vs control group. The data are shown as mean ± SEM (*n* = 5)

The effects of MSG and TA on antioxidant system components were studied at both gene and protein levels. As illustrated in Fig. 3A and B, the marked decrease in *Gpx* mRNA expression caused by MSG was countered by TA treatment. A similar pattern was observed in GPx enzyme activity. The decline in enzyme activity induced by MSG was significantly restored by TA, matching the control group’s levels. *Gst* mRNA expression was notably reduced by MSG exposure, but TA alone did not impact it. Moreover, the combination of MSG and TA significantly mitigated the severe reduction in gene expression compared to MSG alone (Fig. 3C). It was also found that GST enzymatic activity significantly decreased following MSG administration (Fig. 3D). A similar result was observed for glutathione reductase. *Gr* mRNA expression, which was suppressed by MSG exposure alone, rebounded with TA supplementation (Fig. 3E). Additionally, GR enzyme activity was significantly reduced after MSG exposure. However, TA supplementation steadily increased enzyme activity (Fig. 3F). These findings suggest that MSG may increase cellular oxidative stress by suppressing the antioxidant defense system, and TA may significantly alleviate these adverse effects both at the gene level and in terms of enzyme activity, thus renormalizing the cellular redox level.

### Thioredoxin system response in liver tissue after MSG and TA administration

**Fig. 4:**
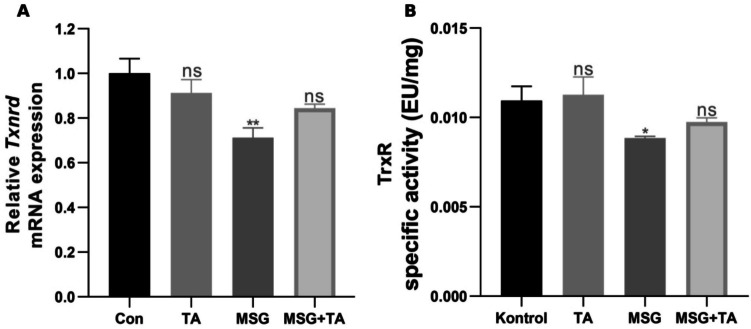
Effects of MSG and TA on the mRNA expression and specific activities of thioredoxin metabolism members in the rat liver tissues. The relative mRNA expressions of *Txnrd*; thioredoxin reductase (**A**). The enzymatic activities of TrxR (**B**) after saline, TA, MSG, and MSG + TA treatment. ns represents *p* > 0.05, *p < 0.05, **p < 0.01, ***p < 0.001, and ****p < 0.0001 vs control group. The data are shown as mean ± SEM (*n* = 5)

Lastly, the levels of thioredoxin reductase mRNA and its enzyme activity were assessed. MSG exposure significantly suppressed *Txnrd* mRNA expression. However, the co-administration of TA with MSG reversed this reduction in gene expression (Fig. 4A). Similar results were observed for thioredoxin reductase activity. Enzyme activity, significantly suppressed by MSG, rebounded with TA treatment (Fig. 4B). These findings indicate that MSG may trigger oxidative stress by suppressing the expression and activity of thioredoxin reductase, an important antioxidant enzyme in the liver. However, TA treatment reverses this suppression, strengthens the antioxidant capacity of the liver, and suggests that it may be an effective candidate for protection against MSG-induced damage.

## Discussion

As the world continues to grow, it is not surprising that the demand for food production will increase. In parallel with the global population growth, food safety and sustainability concerns are increasing (Oluwole et al. 2023). In addition to meeting the increasing demand, food additives, frequently preferred in the food industry to improve the texture of foods and extend their shelf life, raise concerns about their health effects (Muncke et al. 2020). Therefore, to overcome these concerns, it is necessary to determine sustainable food systems that encourage healthy dietary habits. MSG, which is widely used, especially in processed foods today, is an additive that is associated with various health problems. In addition to the metabolic and neurological effects of MSG consumption, it is suggested that it can affect the functions of critical organs, such as the kidneys and liver (Maulina et al. 2024; Subramanian et al. 2023; Yoshida et al. 2024). Recent studies have suggested that MSG consumption may cause changes in liver function, particularly through oxidative stress. Recent studies indicate that TA exhibits potent antioxidant activity through multiple mechanisms (Jing et al. 2022; Tong et al. 2021). TA can protect DNA and proteins from oxidative damage by directly scavenging hydroxyl radicals and reactive oxygen species such as hydrogen peroxide by donating electrons or hydrogen atoms (Wang et al. 2020). In addition, TA upregulates endogenous antioxidant enzymes mostly through the activation of transcription factors such as Nrf2 that bind to antioxidant response elements (ARE) in gene promoters (Jin et al. 2020; Li et al. 2020). TA can further reduce ROS production by inhibiting pro-oxidant enzymes, such as xanthine oxidase and NADPH oxidase (Azimullah et al. 2023). Lastly, TA helps maintain intracellular glutathione balance by reducing its consumption or supporting its regeneration (Laskar et al. 2023). Due to these properties, TA is seen as an ideal candidate to reduce MSG-induced cellular oxidative damage by strengthening antioxidant defenses.

Cells constantly face the challenge of managing oxidants, which can originate from environmental and endogenous factors (Demir et al. 2024; Pizzino et al. 2017). However, their antioxidant networks balance both the production and elimination of reactive oxygen species (ROS). This situation may vary depending on the state of the cell. For example, while ROS elimination is desired for the maintenance of life in healthy cells, the opposite is aimed at in cancer cells to promote ROS production. In this way, the growth and proliferation of tumor cells can be prevented (Nakamura & Takada 2021; Zhou et al. 2014). Glutathione (GSH) and thioredoxin (TRX) systems are essential antioxidant defense mechanisms that maintain cellular redox balance by neutralizing ROS (Ren et al. 2017). The GSH system uses glutathione, a tripeptide composed of glutamate, cysteine, and glycine, as a reducing agent to convert excess ROS into non-toxic compounds, thereby preserving cellular homeostasis (Cassier-Chauvat et al. 2023). The balance between GSH and GSSG is commonly used to assess oxidative stress and the overall redox state within cells, tissues, and bodily fluids. A lower GSH/GSSG ratio indicates heightened oxidative stress, which may lead to a range of diseases and health conditions (Arauz et al. 2016; Nuhu et al. 2020). The TRX system, which includes the redox-active protein Trx and its associated reductase (TrxR), plays a central role in regulating cellular redox balance by catalyzing the reduction of disulfide bonds in target proteins (Drechsel & Patel 2010; Muri & Kopf 2023). As a result, both systems, which are interconnected, ensure tightly regulated redox balance through cross-talk mechanisms. Studies have shown that dysregulation of these antioxidant systems leads to imbalances in several cellular mechanisms, particularly oxidative stress, and contributes to the development of distinct features in various cancers, including hepatocellular carcinoma (HCC) (Abdel-Hamid et al. 2018; Jaganjac et al. 2020; McLoughlin et al. 2019). Recent reports indicate that these systems are upregulated in HCC to help cancer cells cope with oxidative stress induced by their altered metabolism (Dong et al. 2022; Lee et al. 2019). The upregulation of antioxidant systems in cancer cells helps prevent apoptosis and enhances drug resistance, thereby supporting their survival. Furthermore, the increased activity of antioxidant systems in HCC is thought to be a potential therapeutic target. Indeed, inhibiting key components of the GSH and TRX systems, such as GR and TrxR, could increase ROS levels in cancer cells to lethal levels, leading to cell death (Salmain et al. 2023; Xu et al. 2022). However, this suggests that an opposite modulation is required for healthy cells. Control of ROS balance may help protect healthy cells from DNA damage, activation of oncogenic signaling pathways, and mutagenesis, processes that threaten cell viability (Cai et al. 2024). Therefore, fully understanding the roles of the GSH and TRX systems in HCC and identifying the factors leading to their dysregulation is crucial for developing effective therapeutic strategies against liver cancer formation and progression.

In the context of HCC, dysregulation of the GSH and TRX systems is a common adaptive response to abnormal oxidative stress, which disrupts healthy cell metabolism (Brahma et al. 2021). It is thought that the pro-oxidant effect caused by MSG will increase oxidative stress and trigger a faster and more destructive progression of such a scenario. Total glutathione and malondialdehyde levels, which are critical markers of cellular redox balance and are associated with lipid peroxidation, can provide insight into the performance of the antioxidant defense system. Many studies have reported that low GSH and elevated MDA levels correlate with impaired antioxidant metabolism (Chaves et al. 2019; Sahin et al. 2023). In this context, experimental studies conducted with models that have caused hepatic injury with MSG show that MSG causes liver deficits through the disruption of the glutathione system (Moldovan et al. 2023; Shukry et al. 2020).

**Fig. 5:**
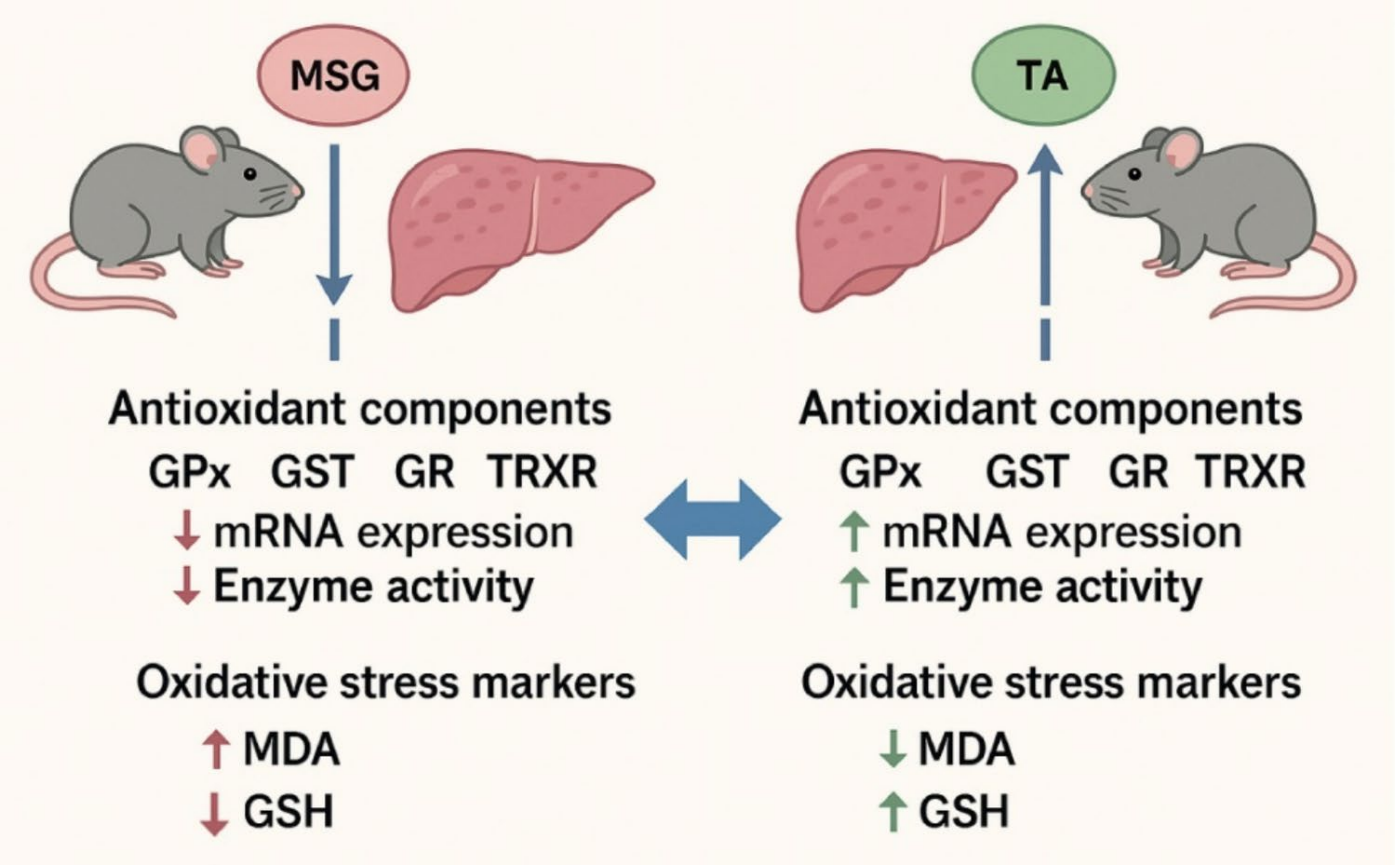
Simplified scheme of the study. Oxidative modulation in liver tissue after MSG exposure and TA treatment. MSG triggers hepatotoxicity by disrupting oxidative modulation in liver tissues. TA restores GST, GPx, GR, and TRXR gene expression and biological activities, promotes lipid peroxidation reduction, and increases glutathione levels

Previous studies have demonstrated acute inhibition or depletion of TrxR sensitizes rat cells to ROS such as hydrogen peroxide (Stancill et al. 2019, 2020). Studies conducted by creating genetic inhibition and/or ablation models for hepatospecific TrxR1 signaling have reported significant alterations in the antioxidant response pathway (Cebula et al. 2015; Suvorova et al. 2009). These previous studies suggest that TrxR is necessary for the cell defense against ROS. When the current literature was examined, no study was found on the MSG-TrxR-liver axis. When evaluated from this perspective, we think that the current study is the first report investigating the effect of MSG on TrxR in liver tissue. The results presented in this study indicate that long-term MSG exposure may disrupt the usual regulation of both GSH and TrxR system components, suppressing ROS neutralization and increasing the likelihood of HCC development (Fig. 5).

## Conclusion

The current tendency in protecting healthy cells against HCC development is towards using treatment strategies such as preventing oxidative stress, which directly affects cell viability. Although the mechanisms behind MSG-induced liver injury are becoming clearer, effectively addressing and mitigating its harmful effects continues to be a major challenge in nutrition-related toxicity, with many aspects still to be uncovered. The results presented in this study show that MSG is an actor that hurts both glutathione and thioredoxin systems. However, further in-depth research, interdisciplinary collaboration, and human studies are vital to improving quality of life, clarifying effective dosages, and optimizing bioavailability. In particular, detailed dose–response investigations are needed to determine the optimal and safe concentrations for biological efficacy. Additionally, studies focusing on the bioavailability, metabolism, and tissue distribution of tannic acid are critical to fully understand its in vivo effectiveness and to guide its potential clinical application.

## Data Availability

All source data for this work (or generated in this study) are available upon reasonable request.
